# Association Between Physical Fitness and Anxiety in Children: A Moderated Mediation Model of Agility and Resilience

**DOI:** 10.3389/fpubh.2020.00468

**Published:** 2020-09-02

**Authors:** Yansong Li, Xue Xia, Fanying Meng, Chunhua Zhang

**Affiliations:** ^1^School of Kinesiology, Shanghai University of Sport, Shanghai, China; ^2^School of Psychology, Shanghai University of Sport, Shanghai, China; ^3^Institute of Physical Education, Huzhou University, Huzhou, China

**Keywords:** children, physical fitness, agility, resilience, anxiety, moderated mediation

## Abstract

**Background:** Anxiety is one of the most prevalent mental health problems in children. Although physical fitness as a predictor of mental health, the mechanisms underlying any association between physical fitness and anxiety in children have been understudied. Thus, the aim of the present study was to determine whether an association exists between physical fitness and anxiety and to explore the roles of agility and resilience in such an association.

**Methods:** This cross-sectional study investigated 269 children aged 7 to 12 years from three public primary schools in Shanghai (China). Physical fitness and agility were objectively measured, and resilience and anxiety were assessed using self-reported questionnaires. The moderated mediation model was examined using the SPSS PROCESS macro, in which the moderator variable was agility, and the mediator variable was resilience.

**Results:** Physical fitness was inversely associated with anxiety. Resilience partially and indirectly mediated this association, and agility moderated the association between physical fitness and resilience. Physical fitness had a greater impact on resilience in children with higher agility levels.

**Conclusions:** Agility moderated the mediation of resilience on the indirect, inverse association between physical fitness and anxiety; thus, incorporating methods to develop agility and resilience may lead to better outcomes for physical fitness programs designed to prevent or alleviate anxiety in children.

## Introduction

Anxiety is one of the most prevalent mental health problems among children (1, 2), with an estimated prevalence up to 20% (3, 4). Anxiety in children has a negative impact on their school performance (5), social functioning (6), and quality of life (7). In addition, anxiety symptoms during childhood tend to be chronic and may lead to anxiety disorders and other serious psychopathological consequences that persist into later childhood and adulthood (1, 8, 9). Indeed, approximately half the anxiety disorders diagnosed in adults have an onset before 11 years of age (10). In addition to the potential long-term effects on individuals' growth and development, children's anxiety disorders place a burden on society via direct and indirect costs (11); public expenses are more than 20 times as high for a child with vs. without clinically relevant anxiety (12). Hence, to mitigate these negative consequences of childhood anxiety, identifying the factors that affect anxiety and their underpinning mechanisms may contribute to the development of effective prevention and treatment interventions to benefit individuals as well as society.

Previous research has shown various protective factors for anxiety, such as self-efficacy (13), coping style (14), and social support (15). A positive role for physical activity in the prevention of anxiety has also been shown in several types of recent studies (16–18). Physical fitness can be defined as the capacity to perform physical activity (19), and physical fitness is more predictive of health outcomes than is physical activity in children (20). Thus, given the established health benefits of physical activity broadly, increasing evidence indicates a need to explore physical fitness. Physical fitness is considered one of the most important health markers and predictors of future risk of mental health issues (21, 22). In addition, physical fitness also serves as a buffer against stress and stress-related disorders (23). Anxiety is a stress-related mental health problem, but the association between physical fitness and anxiety is still unclear (24, 25). Therefore, investigating the mechanisms underlying the role physical fitness plays in childhood anxiety is critically needed.

To date, it is not well-understood which factors are crucial for the maintenance of physical and mental health. Resilience is a relatively new construct that may be an important factor (26). Resilience is the ability to successfully adapt to stress, trauma, or adversity, enabling individuals to avoid stress-induced mental disorders, such as depression, posttraumatic stress disorder, and anxiety (27, 28). In this regard, exploring how to build resilience may be useful in assisting children affected by anxiety. On the other hand, physical fitness may confer resilience owing to its stress-buffering effects (23). Thus, from a stress perspective, physical fitness, and anxiety may be linked through resilience.

In addition, agility, as the main component of physical fitness, is a combination of physical qualities and cognitive components (22, 29). However, unlike studies examining other main components (e.g., cardiorespiratory fitness, muscular fitness), few studies have focused on the contribution of agility to mental health (19). Within the limited literature, agility was summarized as a major input that enables resilience (30). Research has also reported that regular exposure to diverse agility-type movement challenges may facilitate the movement efficiency of athletes and their resilience to numerous dimensions of movement stress (31). Thus, whether the benefits of physical fitness on childhood anxiety may be enhanced by resilience and the role that agility plays in these relationships warrant further attention.

**Figure 1:**
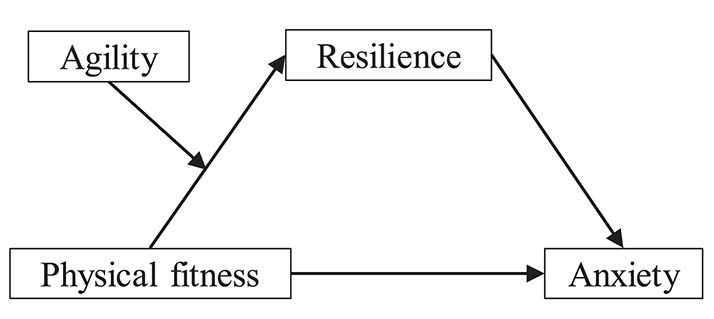
The proposed moderated mediation model to be tested.

Therefore, the present study examined the associations among physical fitness, agility, resilience, and anxiety in children. On the basis of previous research results, we proposed the following three hypotheses: (1) physical fitness is inversely associated with anxiety in children; (2) resilience mediates the association between physical fitness and anxiety; and (3) agility moderates the indirect effect of physical fitness on anxiety through resilience, with the association becoming stronger when agility is high and weaker when agility is low. Based on these hypotheses, we proposed a theoretical model to be tested (Figure 1). In the proposed model, physical fitness plays a role in enhancing resilience and ameliorating anxiety, and the influence of physical fitness on anxiety is mediated by resilience, with the strength of this mediation conditional depending on the level of agility.

## Materials and Methods

### Participants

The participants were selected from three primary schools in Shanghai (China). A sampling of schools was stratified according to physical fitness with three levels, with the first quartile being the lowest, followed by second and third quartiles, and the highest being the fourth quartile, as described and used in previous studies (32, 33). The current three schools were chosen for their representativeness of each level, considering the accessibility to our research team and the availability of the teachers to assist with logistics. Potential participants who had not received a diagnosis of any disease that made it impossible to complete the test and who understood each item in the questionnaires were included. Participants whose physical fitness test results exceeded the logical limits or whose questionnaires were considered invalid were excluded. After the aim of the study was explained, participants completed the objective measurements and self-reported questionnaires under the supervision of trained volunteers. Participants' names were substituted with codes in the data collection to protect privacy, and they were given an inexpensive gift as a token of appreciation. The results obtained from the tests were offered to the school administrators to provide suggestions for participants' physical activity and mental health.

Valid variables were obtained from 269 participants. The participants were in school grades 2 through 5 and comprised 126 boys and 143 girls, with ages ranging from 7–12 years (mean age = 9.75 years; SD = 1.17 years). The number (percentage) of participants by school grade was 72 (26.8%) in second grade, 67 (24.9%) in third grade, 66 (24.5%) in fourth grade, and 64 (23.8%) in fifth grade.

The study was conducted in accordance with the recommendations of the World Medical Association's Declaration of Helsinki as revised in 1989 and was approved by the Shanghai University of Sport Ethics Committee (Shanghai, China). Written informed consent was obtained from the guardians of all participants.

### Measures

#### Physical Fitness

To minimize variability, all physical fitness tests were conducted by trained volunteers from the Shanghai Research Center for Physical Fitness and Health of Children and Adolescents. The volunteers all majored in kinesiology and were already familiar with the testing methods.

Physical fitness was measured using the Chinese National Student Physical Fitness Standard (CNSPFS), a standardized test commonly used in Chinese schools (34). As indicated in the CNSPFS guidelines, the results of different fitness components were scored according to sex and school grade level. The total physical fitness score was composed of the product sum of the scores and weights of each component. The weights of the CNSPFS scores for each fitness component stratified by school grade are given in Table 1.

**Table 1:** Fitness components and weights of CNSPFS scores in school-age children.

**Second grade**		**Third and fourth grades**		**Fifth grade**	
**Fitness component**	**Weight (%)**	**Fitness component**	**Weight (%)**	**Fitness component**	**Weight (%)**
BMI	15	BMI	15	BMI	15
Vital capacity of lung	15	Vital capacity of lung	15	Vital capacity of lung	15
50 m sprint	20	50 m sprint	20	50 m sprint	20
Sit and reach	30	Sit and reach	20	Sit and reach	10
Timed rope-skipping	20	Timed rope-skipping	20	Timed rope-skipping	10
		Timed sit-ups	10	Timed sit-ups	20
				50 m × 8 shuttle run	10

*BMI, Body Mass Index; CNSPFS, Chinese National Student Physical Fitness Standard*.

#### Agility

Agility was assessed using the side-step test, which is an effective and commonly used measure (35). For this test, the participant stood over a central line. At the start of the test, the participant moved laterally, side-stepping toward a far-right line (1 m from the central line) until the right foot crossed the far-right line. Once the right foot crossed that line, the participant changed directions and moved laterally, side-stepping to the left until the left foot crossed the far-left line (1 m from the central line). After reaching the far-left line, the participant moved laterally to return to the central line. This motion was repeated for 20 s, and one point was given for each line passed. The test was conducted twice, and the highest side-step score was recorded.

#### Resilience

Resilience was obtained using the Resilience Scale for Chinese Adolescents (RSCA), which has shown excellent psychometric properties and has been widely used in evaluating the resilience of Chinese children (36). It includes 27 items that have been classified into five factors: target concentration, emotional control, positive thinking, family support, and interpersonal assistance. Participants were asked to rate themselves on questions using a 5-point Likert scale ranging from a score of 1, indicating “not true at all,” to 5, indicating “true nearly all the time.” The total score for the RSCA ranged from 27 to 135, with a higher score indicating a higher level of resilience. Cronbach's alpha for this test in the present study was 0.74.

#### Anxiety

Anxiety was defined using the Chinese version of the Multidimensional Anxiety Scale for Children (MASC), a 39-item self-report scale for assessing children's anxiety (37). The items clustered into the following four scales: physical symptoms, harm avoidance, social anxiety, and separation anxiety. Response options ranged from 0, indicating “never true,” to 3, indicating “often true.” The total score (range, 0–117) was generated by adding the scores of all items; thus, a higher score reflected a greater degree of anxiety. Cronbach's alpha for this scale in this study was 0.89.

#### Statistical Analysis

The data were analyzed using SPSS, version 22.0, and the PROCESS macro program for SPSS (38). Both graphical (normal probability plots) and statistical (Kolmogorov–Smirnov test) methods were used to examine the nature of the variables, and all were found to fit a normal distribution. Thus, parametric statistics were used. Harman's single–factor test was conducted, and the results indicated that no serious method bias existed in the present study.

We conducted descriptive statistics for the main study variables (reported as means ± standard deviations) and Pearson correlation for bivariate associations (reported as values of *r*). We then used the PROCESS macro to perform a regression-based path analysis, which is similar to structural equation modeling but takes into consideration irregular sampling distributions (39). As noted by Edwards and Lambert (40), the distribution of the indirect effect can be non-normal even if the constituent variables are normal. Given this possibility, all regression coefficients were tested using the bias-corrected percentile Bootstrap method (41, 42). We tested the theoretical hypothesis model, controlled for age and sex, by estimating the 95% confidence intervals (CIs) for the mediation and moderation effects, with 5,000 resampled samples. When a 95% CI did not include 0, the result was considered statistically significant. We selected the Model 4 in PROCESS to examine the simple mediation effect of resilience on the association between physical fitness and anxiety. We then incorporated the proposed moderator variable (agility) into the model by conducting moderated mediation (also known as a conditional indirect effect) analysis using the Model 7 in PROCESS to determine whether the indirect path was conditionally moderated by the “strength” or value of agility. This model uses ordinary least squares regression to assess the conditional indirect effect and tests the effect with bootstrap CIs for different values of the moderating variable (agility) to determine whether the indirect effect varies. Before formal data analysis, all variables were standardized. Values of *p* < 0.05 were considered statistically significant.

## Results

Descriptive statistics and correlations of the study variables are shown in Table 2. We found that physical fitness, agility, and resilience were inversely correlated with anxiety; physical fitness was directly correlated with agility and resilience; and agility was directly correlated with resilience.

**Table 2:** Descriptive statistics and correlations between variables.

**Variable**	**Mean ± SD**	**1**	**2**	**3**	**4**
(1). Physical fitness	81.50 ± 9.80	–			
(2). Agility	37.18 ± 9.03	0.408^***^	–		
(3). Resilience	93.10 ± 13.77	0.248^***^	0.267^***^	–	
(4). Anxiety	43.33 ± 19.18	−0.244^***^	−0.154^*^	−0.247^***^	–

*SD, standard deviation*;

* *p < 0.05*,

*** *p < 0.001*.

As shown in Table 3, the results of simple mediation model testing using Model 4 indicated that physical fitness was directly associated with resilience (β = 0.249, *t* = 4.139, *p* < 0.001). Resilience was inversely associated with anxiety (β = −0.190, *t* = −3.145, *p* < 0.01). The association between physical fitness and anxiety was also significant (β = −0.208, *t* = −3.423, *p* < 0.01). We also found a significant indirect effect of physical fitness on anxiety via resilience: the bootstrapping results indicated an indirect effect [β = −0.047; 95% CI: (−0.088, −0.013)]. The indirect effect accounted for 18.4% of the total effect, suggesting that resilience played a partial mediating role in the association between physical fitness and anxiety. These findings supported our first and second study hypotheses.

**Table 3:** Mediation modeling results assessing the effect of physical fitness on anxiety.

**Effect**	**Path**	**β**	**SE**	***t***	**LLCI**	**ULCI**
Total effect	Physical fitness–anxiety	−0.256	0.060	−4.261^***^	−0.374	−0.137
Direct effect	Physical fitness–resilience	0.249	0.060	4.139^***^	0.131	0.367
	Resilience–anxiety	−0.190	0.060	−3.145^**^	−0.308	−0.071
	Physical fitness–anxiety	−0.208	0.061	−3.423^**^	−0.328	−0.089
Indirect effect	Physical fitness–resilience–anxiety	−0.047	0.019	-	−0.088	−0.013

*β, standardized coefficients; SE, standard error; LLCI and ULCI, lower level and upper level of the bias-corrected 95% bootstrap confidence interval*;

** *p < 0.01*,

*** *p < 0.001*.

**Figure 2:**
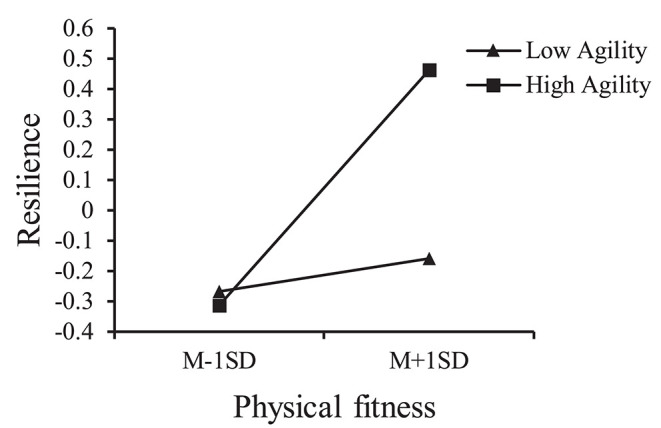
Agility moderated the effect of physical fitness on resilience.

Moderated mediation analysis using Model 7 was conducted to assess whether anxiety was indirectly affected by physical fitness via mediation through resilience and whether this effect was conditionally moderated by agility. We hypothesized that children with higher agility scores would show a stronger association between physical fitness and resilience compared with those with lower agility scores. Table 4 shows the results of the moderated mediation model test. The effect of the interaction between physical fitness and agility on resilience was statistically significant (β = 0.167, *t* = 2.597, *p* < 0.05). Figure 2 illustrates the interaction at high (plus 1 SD) and low (minus 1 SD) levels of physical fitness and agility. The plots indicate the interaction between physical fitness and agility on resilience and suggested that for children with higher agility levels, there was a stronger positive association between physical fitness and resilience compared with children having lower agility levels.

**Table 4:** Moderated mediation modeling results assessing the effect of physical fitness on anxiety.

**Outcome variable**	**Factor**	**β**	**SE**	***t***	**LLCI**	**ULCI**	***R^**2**^***	***F***
Resilience	Physical fitness	0.222	0.067	3.312^**^	0.090	0.353	0.119	7.129^***^
	Agility	0.144	0.068	2.119^*^	0.010	0.278		
	Physical fitness × agility	0.167	0.064	2.597^*^	0.040	0.293		
	Age	0.045	0.052	0.859	−0.058	0.147		
	Sex	−0.052	0.118	−0.444	−0.283	0.179		
Anxiety	Physical fitness	−0.208	0.061	−3.423^**^	−0.328	−0.089	0.109	8.061^***^
	Resilience	−0.190	0.060	−3.145^**^	−0.308	−0.071		
	Age	−0.052	0.050	−1.031	−0.150	0.047		
	Sex	0.187	0.118	1.586	−0.045	0.419		

*β, standardized coefficients; SE, standard error; LLCI and ULCI, lower level and upper level of the bias-corrected 95% bootstrap confidence interval*;

* *p < 0.05*,

** *p < 0.01*,

*** *p < 0.001*.

The conditional indirect effect of physical fitness on anxiety through resilience at various values of agility was analyzed when the agility score was the sample mean and also at plus or minus 1 SD. The results revealed that the conditional indirect effect was significant at the mean and at a high level (plus 1 SD) of the moderator (agility), but not at a low level (minus 1 SD), and the bootstrap 95% CIs supported these results (see Table 5). In addition, the moderated mediation index was also statistically significant. These results supported our third study hypothesis because the association between physical fitness and the outcome variables became more evident as the moderator value increased.

**Table 5:** Moderated mediation effect of physical fitness on anxiety at specific conditional values of agility.

	**Specific conditional values of agility**			
	**β**	**SE**	**LLCI**	**ULCI**
−1 SD	−0.010	0.015	−0.046	0.017
Mean	−0.042	0.017	−0.077	−0.010
+1 SD	−0.074	0.030	−0.134	−0.017
	**Index of moderated mediation**			
	**Index**	**SE**	**LLCI**	**ULCI**
Agility	−0.032	0.016	−0.066	−0.003

*β, standardized coefficients; SD, standard deviation; SE, standard error; LLCI and ULCI, lower level and upper level of the bias-corrected 95% bootstrap confidence interval*.

## Discussion

In the present study, we used a moderated mediation model analysis to assist in understanding the mechanisms underlying any association between physical fitness and anxiety in children. Our results supported our three study hypotheses, namely, that (1) higher physical fitness levels were associated with lower anxiety levels in children, (2) resilience mediated physical fitness and anxiety, and (3) agility moderated the mediation of resilience on the association, which was stronger in children with higher levels of agility.

Our results suggested that higher levels of physical fitness were associated with lower anxiety levels in children. A plausible explanation for this finding is that individuals who display higher levels of physical fitness tend to have more positive perceptions of anxiety symptoms and thus feel less anxious (25). This supposition is supported by several studies that have shown that improvements in physical fitness are associated with reductions in levels of anxiety (43–45). However, Rodriguez-Ayllon et al. (19) found no relationship between physical fitness components and anxiety indicators. In addition to the demographic characteristics of the participants, these discrepant results may be because those previous studies focused on specific components of physical fitness (46), but physical fitness components relate in different ways to the various aspects of mental health (19). Our study results were based on the total physical fitness scores of children obtained by CNSPFS. This comprehensive outcome reflects the basic physical fitness of children and is an important basis for judging the overall level of physical fitness in children. Further analysis of physical fitness as a protective factor may provide a new approach for the prevention or intervention of childhood anxiety. Moreover, the fitness components, standards for evaluation, and score weights vary in the CNSPFS to adjust for the growth and development of children. Thus, incorporating interventions to reduce the level of anxiety through physical fitness programs that are appropriate at each age in children may lead to better outcomes.

Our mediation model revealed that resilience mediated the association between physical fitness and anxiety, indicating that establishing good physical fitness was conducive to resilience in children, which in turn ameliorates their anxiety. Physical fitness appears to confer resilience by blunting or optimizing neuroendocrine and physiological responses (e.g., the hypothalamic-pituitary-adrenal axis, the sympathetic nervous system) to physical and psychosocial stressors (23). In line with previous studies (47–49), our result is meaningful in that it supports physical fitness being an effective manner of building resilience in children. On the other hand, resilience can also indirectly reduce the negative effects of stress on anxiety through its mediation effect (50, 51). As a dynamic process, resilience is construed as an active adaptation mechanism that may help alleviate anxiety (27), which was observed in our study. In other words, resilience accounting for an important part of the variations in anxiety symptoms suggests that interventions should target ways to enhance resilience (50).

We speculate that a biological mechanism that might provide an explanation for our findings is that physical fitness may promote resilience by minimizing inflammation. The benefits of physical fitness may, in part, be attributed to anti-inflammatory effects via changes in body composition and skeletal muscle (23). On the other hand, Interleukin-8 as an inflammatory marker, has been suggested to be involved in the biological mechanisms mediating resilience to anxiety (52). Overall, resilience may serve as a “bridge” linking physical fitness and anxiety; thus, it would be insufficient to attempt to reduce anxiety in children by promoting physical fitness alone because resilience also plays an important role.

Our moderated mediation model offered a more detailed picture of the mechanisms that associated physical fitness with anxiety, providing a basis for improving anxiety in children using physical fitness via full consideration of the characteristics of agility. However, in research investigating the relationship between physical fitness and mental health, to the best of our knowledge, there is no in-depth analysis examining the importance of agility. Therefore, in our study, we used agility as an independent moderator to assess its influence. Interestingly, we found that physical fitness did not have a significant effect on resilience when the level of agility was low, but the positive effect of physical fitness occurred with the improvement of agility. More importantly, these results were confirmed in our physical fitness–anxiety model. These findings support our hypothesis that in relation to mental health, agility plays a critical role in the effects of physical fitness.

The self-efficacy based model of resilience suggests that executive function provides much of the capacity for resilience in individuals, with executive function enhancing self-efficacy to enable successful adjustment (53, 54). Additionally, executive function yields a greater propensity for resilience in children (55, 56). On the other hand, recent evidence suggests that agility is a key component for executive function, and children with higher levels of agility have shown better performance in executive function (46, 57). Taken together, these findings indicate that executive function combines agility with resilience—which to some extent reflects the adaptation and coping ability of children (56)—to help explain the pathway between agility and resilience and provide a new perspective for further understanding how physical fitness affects resilience. More interestingly, the side-step test used in the present study is also considered useful for improving cardiorespiratory and muscular fitness (35). These main fitness components may all contribute to the promotion of resilience (19, 23). Therefore, the potential connections appear to demonstrate the value of agility, which should be taken into account for physical fitness to benefit resilience. To gain greater insight into the moderation effect of agility on the association between physical fitness and anxiety, both theoretical and empirical research studies are needed in the future.

Our study has some limitations that should be considered when interpreting our results. First, this study used a cross-sectional design, which prevented us from making any cause-and-effect conclusions. Therefore, longitudinal studies should be designed to validate the findings. Second, our study relied in part on self-reported data, which may affect the outcomes. Future studies should measure mental health objectively, such as through neuropsychological tests. Third, our findings were obtained from children in public primary schools, and whether the results are applicable to other populations will require further testing.

## Conclusions

The current study offers some insight into the mechanisms underlying the association between physical fitness and anxiety. Physical fitness is a valuable factor, providing some protection against anxiety for children. This protective effect is partially mediated through the resilience, which is moderated by agility. The moderation effect may enable children with higher agility levels to show better outcomes in the association between physical fitness and anxiety. The analysis conducted in the present study using a moderated mediation model analysis expands on the current knowledge by providing evidence to support potential mechanisms. The results of this analysis suggest that incorporating methods to develop agility and to improve resilience may lead to better outcomes when designing physical fitness programs to prevent or alleviate anxiety in children.

## Data Availability Statement

The raw data supporting the conclusions of this article will be made available by the authors, without undue reservation.

## Ethics Statement

The studies involving human participants were reviewed and approved by Shanghai University of Sport Ethics Committee. Written informed consent to participate in this study was provided by the participants' legal guardian/next of kin.

## Author Contributions

YL, FM, and CZ contributed to the conception and design of the study. XX and FM organized the database. YL and XX performed the statistical analysis and wrote the first draft of the manuscript. CZ revised the manuscript. All authors contributed to manuscript revision, read, and approved the submitted version.

## Conflict of Interest

The authors declare that the research was conducted in the absence of any commercial or financial relationships that could be construed as a potential conflict of interest.
